# Jazf1 promotes prostate cancer progression by activating JNK/Slug

**DOI:** 10.18632/oncotarget.23146

**Published:** 2017-12-12

**Authors:** Yonghun Sung, Song Park, Si Jun Park, Jain Jeong, Minjee Choi, Jinhee Lee, Wookbong Kwon, Soyoung Jang, Mee-Hyun Lee, Dong Joon Kim, Kangdong Liu, Sung-Hyun Kim, Jae-Ho Lee, Yun-Sok Ha, Tae Gyun Kwon, Sanggyu Lee, Zigang Dong, Zae Young Ryoo, Myoung Ok Kim

**Affiliations:** ^1^ School of Life Science, BK21 Plus KNU Creative Bio Research Group, College of Natural Sciences, Kyungpook National University, Buk-ku, Daegu, Republic of Korea; ^2^ Core Protein Resources Center, Daegu Gyeongbuk Institute of Science and Technology (DGIST), Daegu, Republic of Korea; ^3^ China-US (Henan) Hormel Cancer Institute, Zhengzhou, Henan, China; ^4^ Department of Anatomy, Keimyung University School of Medicine, Dalseo-gu, Daegu, Republic of Korea; ^5^ Department of Urology, Kyungpook National University Medical Center, Buk-gu, Daegu, Korea; ^6^ The Hormel Institute, University of Minnesota, NE, Austin, Minnesota, USA; ^7^ The School of Animal BT Science, Kyungpook National University, Sangju-si, Gyeongsangbuk-do, Korea

**Keywords:** Jazf1, prostate cancer, metastasis, JNK, slug

## Abstract

Juxtaposed with another zinc finger protein 1 (Jazf1) is a zinc finger protein and is known to affect both prostate cancer and type 2 diabetes. Jazf1 inhibits testicular nuclear receptor 4 (TR4) activation through protein-protein interaction, which results in weight loss and alleviates diabetes. However, the role of Jazf1 in prostate cancer is still poorly understood. Hence, we investigated whether the expression of Jazf1 is associated with prostate cancer progression. We confirmed the upregulation of Jazf1 expression in human prostate tissue samples. In addition, using Jazf1 overexpressing prostate cancer cell lines, DU145 and LNCaP, we found Jazf1 promoted cell proliferation and colony formation ability. We also observed that Jazf1 dramatically enhanced cell migration and invasion in transwell assays. Additionally, we checked the upregulation of vimentin and downregulation of E-cadherin expression in Jazf1-overexpressing DU145 and LNCaP cells. Moreover, we found that Slug, which is known to be regulated by JNK/c-Jun phosphorylation, was upregulated in the microarray analysis of two prostate cancer cell lines. Jazf1 promotes the phosphorylation of JNK/c-Jun, likely promoting cell proliferation and invasion through Slug. In a xenograft model, tumors overexpressing Jazf1 were larger than control tumors, and tumors with decreased Jazf1 were smaller. These data indicated that Jazf1 enhances prostate cancer progression and metastasis via regulating JNK/Slug signaling. Taken together, these results suggest that Jazf1 plays an important role in both androgen dependent and independent prostate cancer.

## INTRODUCTION

Prostate cancer is the most diagnosed cancer and the second leading cause of cancer-related death in men from developed countries [1–3]. Most prostate cancers rely on the secretion of hormones, especially androgen, for cell survival, hence, hormone deprivation therapies inhibit prostate cancer progression [2, 4, 5]. However, prostate cancers treated with hormone therapy eventually recur as castration-resistant prostate cancer [6]. Castration-resistant prostate cancer is implicated in metastasis to the bone, and responds poorly to chemotherapy [7–9]. Most prostate cancer-related deaths occur from castration resistance [9]. Therefore, it is important to understand molecular mechanisms of prostate cancer, including androgen-dependent mechanisms, to decrease prostate cancer-related deaths.

Juxtaposed with another zinc finger protein 1 (Jazf1) is composed of three putative zinc finger motifs, which encode a cysteine-2 histidine-2 (Cys2-His2) zinc finger protein that interacts with heavy metals *in vitro* [10]. Jazf1 represses testicular nuclear receptor 4 (TR4) through protein-protein interaction [11]. TR4 activates gluconeogenesis by promoting PEPCK transcription, and causes weight gain and body fat accumulation [12, 13]. Jazf1 as a TR4 repressor decreases the expression of PEPCK and inhibits body weight gain in a high fat diet *in vivo* [14]. Additionally, Jazf1 is associated with tumor progression, including endometrial stromal sarcoma and prostate cancer [15–18]. A Jazf1-SUZ12 fusion protein inhibits PRC2 complexes that disturb chromatin formation in endometrial stromal sarcoma [19]. A variety of evidence indicates a correlation between Jazf1 and prostate cancer [15–17]. Numerous studies have shown that Jazf1 affects both diabetes and prostate cancer risk, and is highly expressed in aggressive prostate cancer [15–17]. However, the molecular mechanism of Jazf1 has not yet been clarified.

Slug is a member of the Snail superfamily, a conserved C2H2-type zinc finger transcription factor, and is well-known as an epithelial-mesenchymal transition (EMT) factor [20, 21]. Slug enhances EMT and metastasis, including cell migration and invasion, by repressing E-cadherin in various cancers such as breast, lung, and prostate [21–23]. In the case of prostate cancer, the Slug/E-cadherin pathway promotes cancer progression through p19Arf in a mouse model [24]. A recent study also showed that Slug promotes prostate tumorigenesis by directly repressing the tumor suppressor PTEN [23]. Slug expression is increased by the phosphorylation of JNK, through increased c-Jun expression [24]. Both Slug and Jazf1 promote prostate cancer tumorigenesis, but the relationship between them is still unknown.

This study used human prostate cancer cell lines to observe cellular activities and molecular mechanisms related to the progression and invasiveness of prostate cancer. We found that Jazf1 promoted prostate cancer cell proliferation and invasion by increasing the subsequent expression of JNK and Slug. Additionally, we observed that Jazf1 is expressed higher in human prostate cancer tissues than in normal tissue. These results suggest that the Jazf1/Slug axis contributes to prostate cancer progression and is a potential anti-cancer target.

## RESULTS

### Expression of Jazf1 is enhanced in human prostate cancer tissues

**Figure 1:**
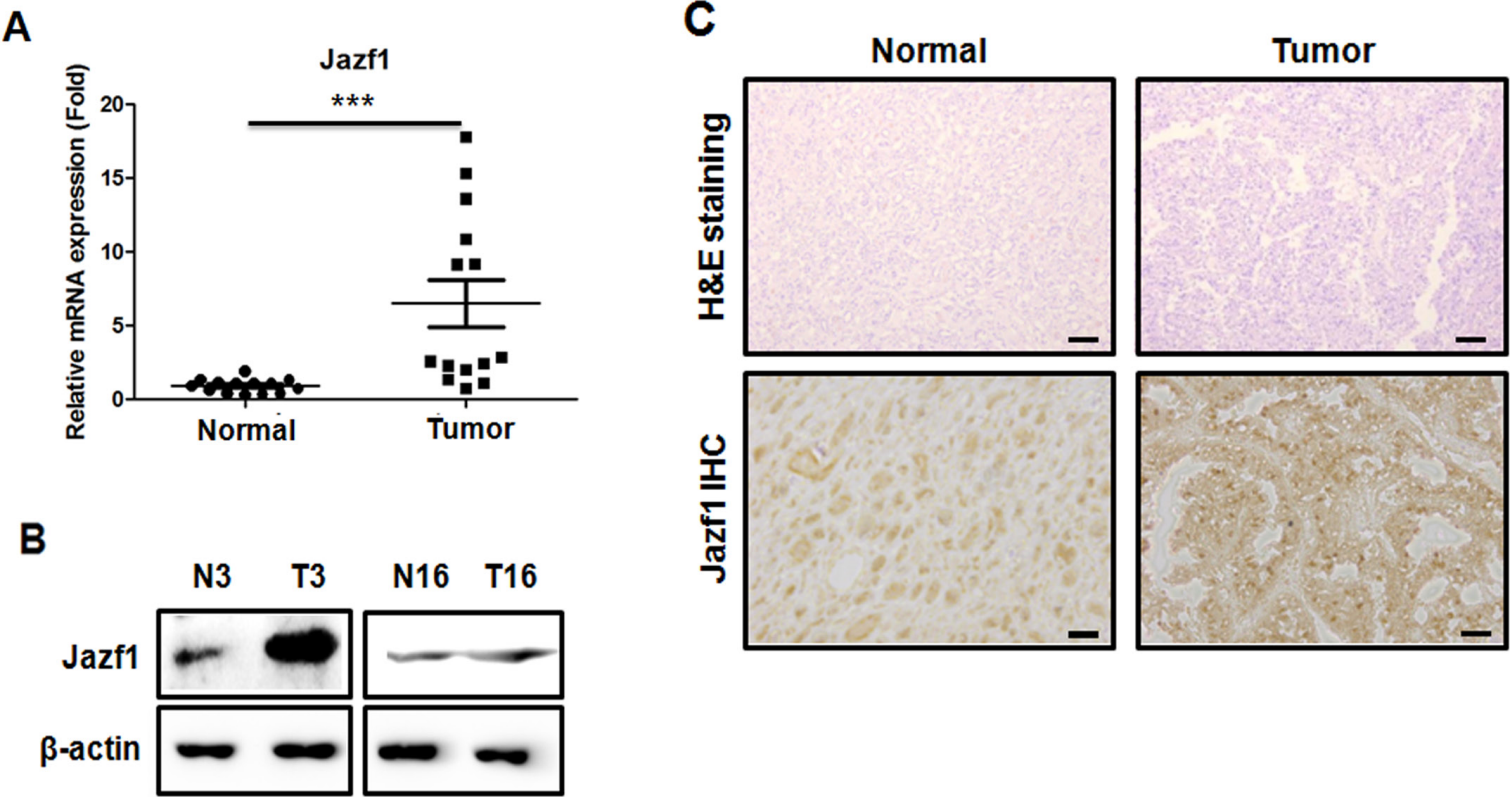
Analysis of Jazf1 expression in human prostate tissue (**A**) Relative expression of *Jazf1* mRNA was measured in human prostate tissues. (**B**) Jazf1 protein levels were compared between prostate tumor tissues (T3, T16) and adjacent normal prostate tissues (N3, N16). (**C**) H&E staining (50× magnification, scale bar = 100 µm) and anti-Jazf1 (100× magnification, scale bar = 100 µm) were performed in human prostate tissues (Means ± SD, ^***^p < 0.001, compared with control).

Recent reports showed that the Jazf1 gene is associated with prostate cancer risk [15–17], so we compared Jazf1 expression between human normal prostate and prostate cancer tissues. The levels of Jazf1 mRNA (Figure 1A) and protein (Figure 1B) were increased in human prostate cancer tissues compared to adjacent normal prostate tissues. Next, we performed immunohistochemistry in human tissues. The expression of Jazf1 in prostate cancer tissues was higher than in normal tissues (Figure 1C). Together, Jazf1 was higher expression in human prostate cancer tissues, suggesting Jazf1 may be linked to prostate cancer development.

### Jazf1 expression alters cell proliferation and colony formation in prostate cancer cells

**Figure 2:**
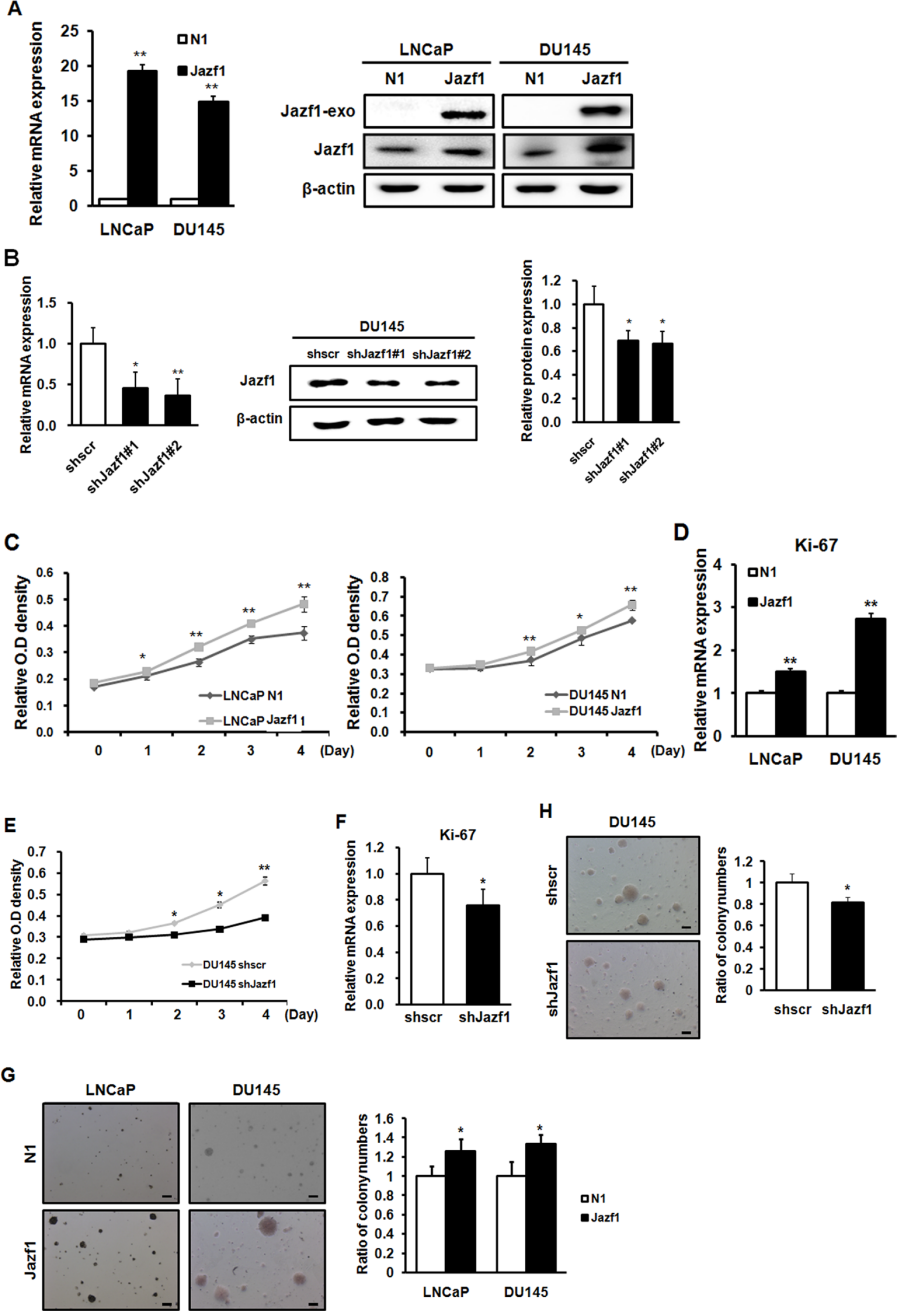
Jazf1 enhances cell proliferation and colony formation ability in human prostate cancer cells (**A**) Jazf1-overexpressing human prostate cancer cell lines were established. The mRNA and protein levels of Jazf1 were measured upon Jazf1 overexpression. (**B**) A Jazf1 knock-down DU145 cell line was established. The mRNA and protein levels of Jazf1 were measured in both scrambled and Jazf1 knock-down DU145 cells. (**C**) The relative O.D. values of cell lines were measured in Jazf1-overexpressing cell lines using a CCK-8 assay. (**D**) The relative mRNA expression of *Ki-67* was examined in Jazf1-overexpressing cell lines. (**E**) Relative O.D. values of Jazf1 knock-down DU145 cells were compared with scrambled DU145 cells. (**F**) The mRNA expression of *Ki-67* was measured in Jazf1 knock-down DU145 cells. (**G**) Colony formation ability in Jazf1-overexpressing cell lines were compared with empty vector cell lines (50× magnification, scale bar = 100 µm). The numbers of colonies were counted in Jazf1-overexpressing cell lines. (**H**) Colony formation ability in Jazf1 knock-down DU145 cells was compared to that of scrambled DU145 cells (50× magnification, scale bar = 100 µm). The numbers of colonies were counted in Jazf1 knock-down DU145 cells. N1; pEGFP-N1 empty vector, shscr; shscrambled vector (Means ± SD, ^*^p < 0.05, ^**^p < 0.01 compared with control).

Based on the human data, we performed cellular activity experiments using human prostate cancer cell lines LNCaP and DU145. To investigate the effects of Jazf1 in prostate cancer, we established prostate cancer cell lines stably overexpressing Jazf1. The mRNA and protein levels of Jazf1 were higher in Jazf1-overexpressing cells than in empty vector-expressing cells (Figure 2A). Next, we established stable Jazf1 knock-down in DU145 cells. We identified decreased expression in Jazf1 knock-down cells using qRT-PCR and western blot analyses (Figure 2B). To measure cancer cell proliferation, we performed a CCK-8 proliferation assay. Proliferation was examined in Jazf1-overexpression cell lines and Jazf1 knock-down DU145 cells at 0, 1, 2, 3, and 4 days. Jazf1 overexpression enhanced cell proliferation in LNCaP and DU145 cell lines, and decreased expression of Jazf1 suppressed proliferation in DU145 cells (Figure 2C, 2E). In addition, the mRNA levels of Ki-67, a proliferation marker, were increased in Jazf1-overexpressing cell lines compared to control cell lines, and levels decreased in Jazf1 knock-down DU145 cells (Figure 2D, 2F). These data suggest that Jazf1 promotes prostate cancer cell proliferation. In addition, we measured cells’ colony-forming ability through soft agar assays. Colony number and diameter were enhanced in Jazf1-overexpressing cells and decreased in knock-down cells (Figure 2G, 2H). These findings suggest that Jazf1 plays a critical role in prostate cancer progression by promoting proliferation and colony formation ability.

### Jazf1 activates cell migration and invasiveness, regulating EMT markers

**Figure 3:**
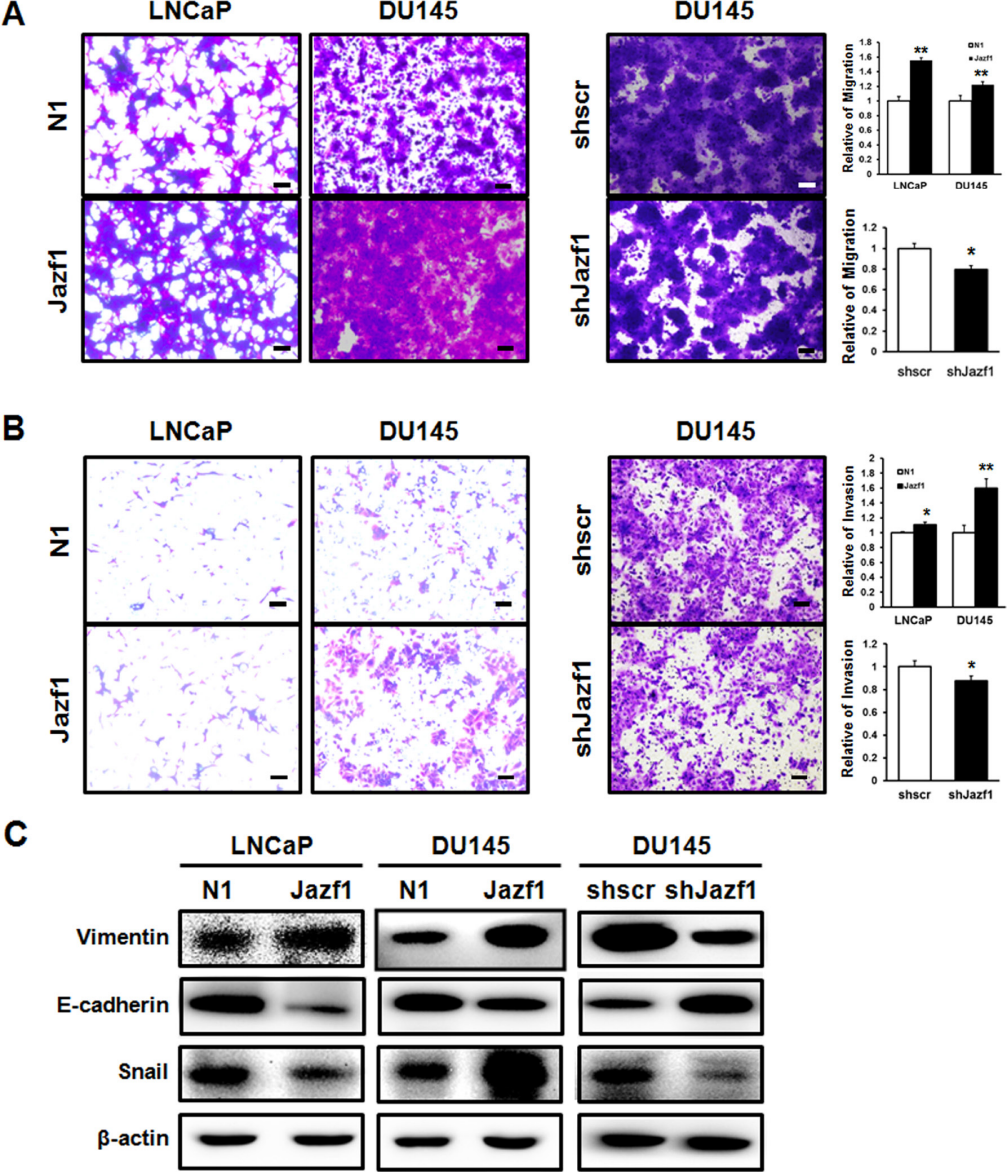
Jazf1 increases migration and invasion abilities (**A**) Migrated cells were stained with crystal violet in both Jazf1-overexpressing and knock-down cell lines (50× magnification, scale bar = 100 µm). (**B**) Cells that invaded through Matrigel were stained with crystal violet in both Jazf1-overexpressing and knock-down cell lines (50× magnification, scale bar = 100 µm). (**C**) The protein levels of EMT markers were measured in both Jazf1-overexpressing and knock-down cell lines using western blot. β-actin was measured as control. N1; pEGFP-N1 empty vector, shscr; shscrambled vector (Means ± SD, ^*^p < 0.05, ^**^p < 0.01 compared with control).

To investigate cell mobility and invasion, we performed migration and invasion assays using transwell assays. Migration ability in Jazf1-overexpressing cell lines was enhanced compared to migration of control cell lines (Figure 3A). In Jazf1 knock-down cells, cell migration was decreased compared to migration of the scrambled cell line (Figure 3A). In invasion assay, the cells that invaded through Matrigel were measured in LNCaP and DU145 cell lines. The number of invaded cells was significantly increased in Jazf1-overexpressing cells compared to that of control cell lines. Jazf1 knock-down cells exhibited decreased invasive capacity than scrambled cells (Figure 3B). Next, we measured EMT marker levels using western blot analysis. Levels of vimentin, a mesenchymal cell marker, and Snail were increased in Jazf1-overexpressing cells and decreased in Jazf1 knock-down DU145 cells. On the other hand, expression of E-cadherin, an epithelial cell marker, was decreased by Jazf1 expression (Figure 3C). Taken together, these results suggest that Jazf1 promotes metastasis and EMT through enhanced migration and invasion, increasing mesenchymal cell markers, and decreasing epithelial cell markers.

### Jazf1 promotes Slug expression through JNK signaling pathway

**Figure 4:**
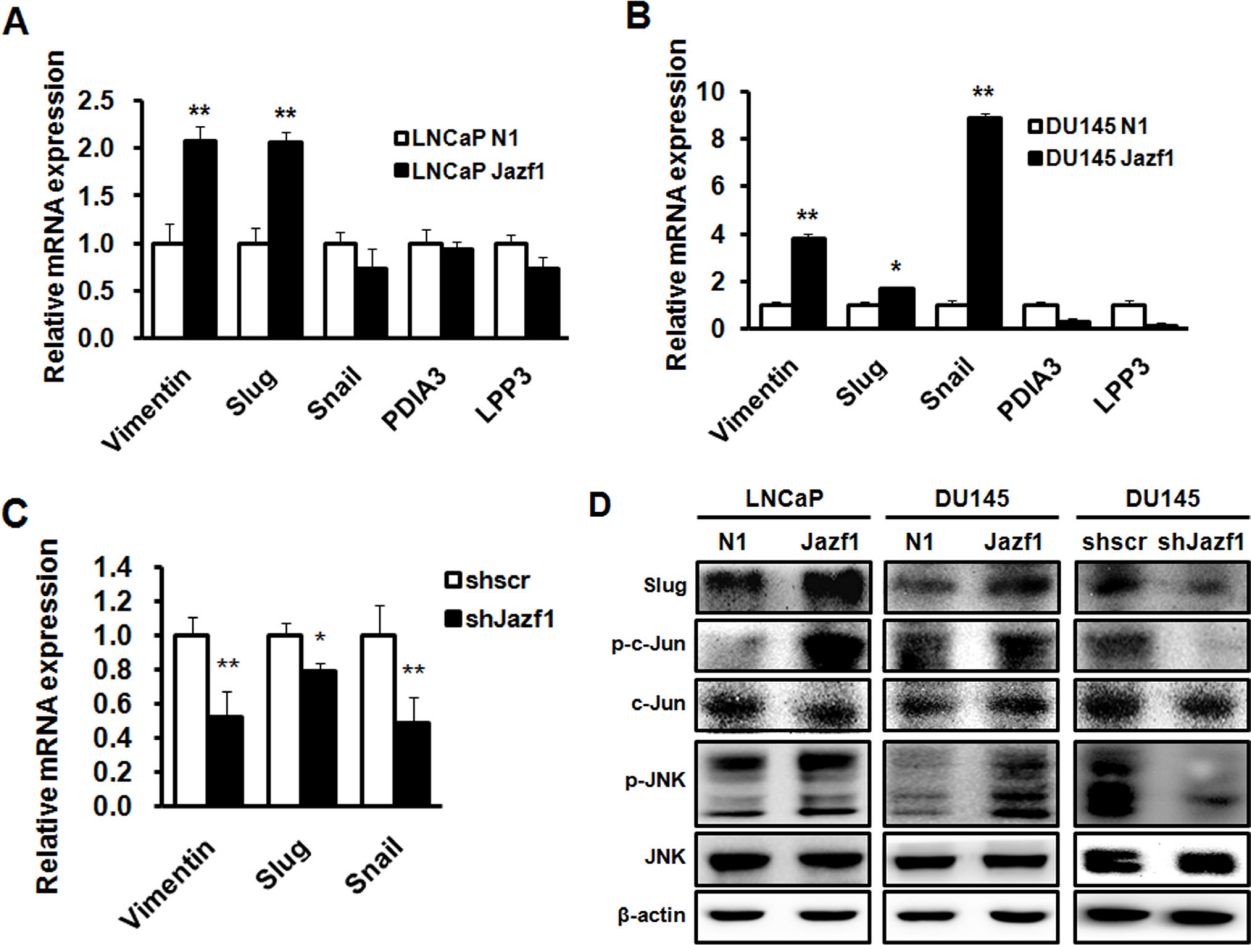
Jazf1 promotes Slug expression through JNK/AP-1 signaling (**A**, **B**) Relative mRNA expression of five candidate genes based on microarray was measured using qRT-PCR. (**C**) Relative mRNA expression of three genes was measured in Jazf1 knock-down DU145 cells. (**D**) Protein levels of Slug and JNK/c-Jun phosphorylation were examined using western blotting in both Jazf1-overexpressing and knock-down cells. β-actin was measured as control. N1; pEGFP-N1 empty vector, shscr; shscrambled vector (Means ± SD, ^*^p < 0.05, ^**^p < 0.01, compared with control).

To assess downstream signaling of Jazf1, we performed a microarray in prostate cancer cell lines. In microarray data, levels of five genes were monitored in both LNCaP and DU145 cells. We measured the mRNA expression of five genes in LNCaP and DU145 cells using qRT-PCR. Both vimentin and Slug were significantly increased in Jazf1-overexpressing prostate cancer cell lines (Figure 4A, 4B). In addition, Jazf1 knock-down DU145 cells exhibited decreased expression of vimentin and Slug (Figure 4C). The protein level of Slug was significantly altered according to the expression levels of Jazf1 (Figure 4D). Previous studies showed that Slug accelerates prostate cancer progression and metastasis through the JNK/c-Jun signaling pathway [24]. Therefore, we explored the phosphorylation of JNK and c-Jun through western blot analysis. Jazf1 overexpression induced the phosphorylation of JNK and c-Jun in LNCaP and DU145 cell lines, and phosphorylation of these proteins declined upon Jazf1 knock-down (Figure 4D). These data demonstrated that Jazf1 enhances Slug expression by activating JNK phosphorylation, suggesting that Jazf1 promotes prostate cancer progression.

### Jazf1 regulates tumor cell tumorigenicity *in vivo* in prostate cancer

**Figure 5:**
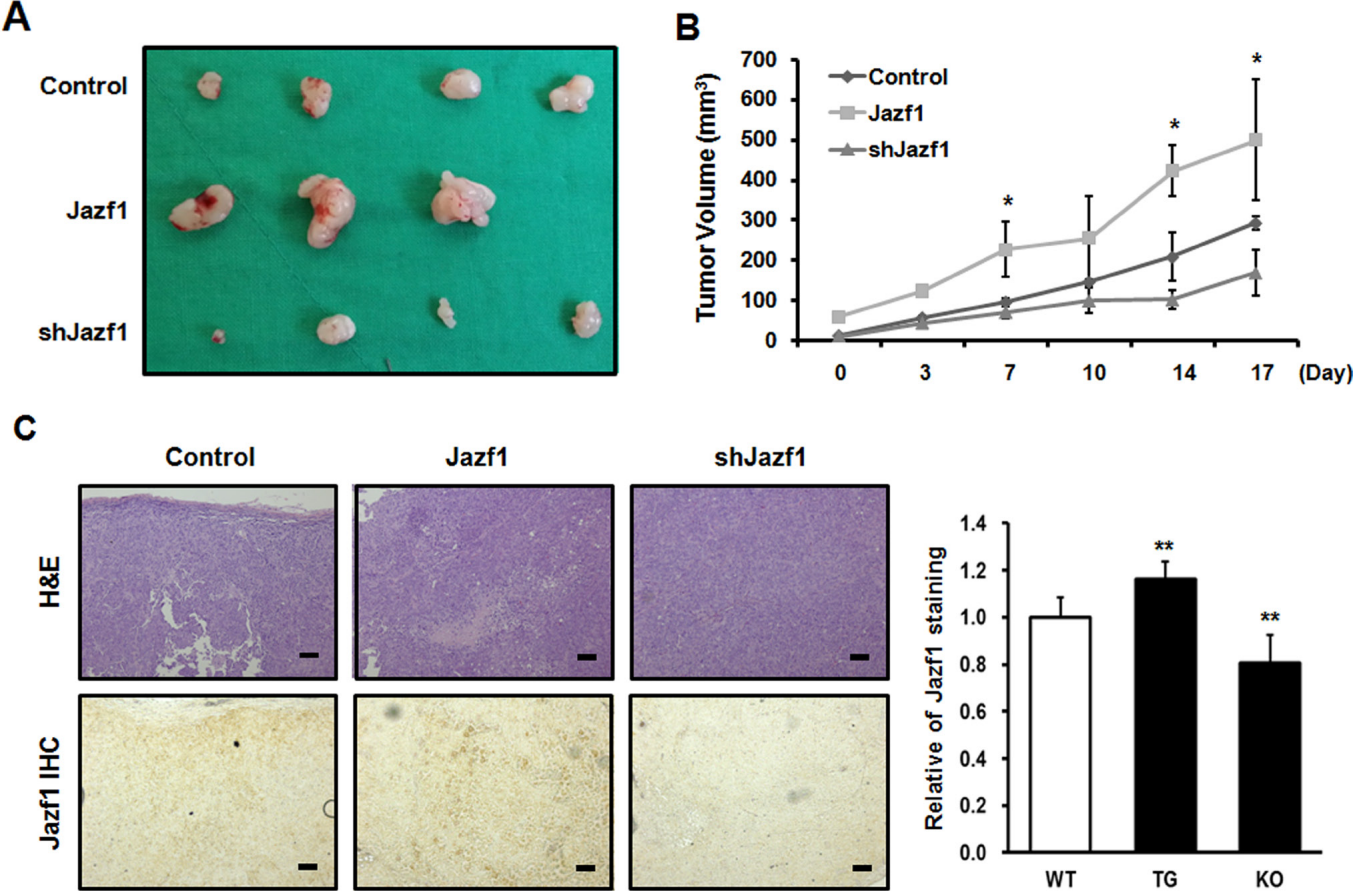
Jazf1 regulates tumor formation in nude mice (**A**) Jazf1-overexpressing or knock-down DU145 cells were subcutaneously injected in nude mice and representative tumor tissues were observed after 5 weeks. (**B**) Tumor volumes were measured twice a week for 3 weeks after tumor formation. (**C**) Immunohistochemistry (IHC) was performed on tumors. Both H&E and anti-Jazf1 were stained in control, Jazf1 overexpression, and knock-down tumor samples (50× magnification, scale bar = 100 µm). N1; pEGFP-N1 empty vector, shscr; shscrambled vector (Means ± SD, ^*^p < 0.05, compared with control).

To test the role of Jazf1 in tumor formation *in vivo,* DU145 cells were subcutaneously injected into athymic nude mice and tumor growth was monitored for 5 weeks. During 5 weeks, Jazf1-overexpressing tumors were larger than control tumors, and Jazf1 knock-down tumors were smaller (Figure 5A, 5B). We performed immunohistochemistry (IHC) on tumor tissues. All tumor tissues were also subjected to H&E staining (Figure 5C). Jazf1 expression was higher in Jazf1-overexpressing cells and lower in Jazf1 knock-down cells compared to control cells (Figure 5C). These findings suggest that elevated expression of Jazf1 promotes prostate tumorigenesis *in vivo*.

## DISCUSSION

Various reports have shown that Jazf1 is associated with a variety of diseases, including cardiac disease, diabetes, endometrial stromal sarcoma, and prostate cancer [15, 18, 25, 26]. Previously, Jazf1 was mainly researched in the context of diabetes, because Jazf1 is a novel repressor of TR4, a kind of nuclear receptor that regulates gluconeogenesis [25, 27]. Accordingly, recent studies reported that Jazf1 also regulates gluconeogenesis and lipid metabolism [14, 25]. Jazf1 inhibits the abilities of TR4 as transcription regulator, thereby suppressing gluconeogenesis [11]. In addition, Jazf1 affects human cancers, especially prostate cancer and endometrial stromal sarcomas [18, 28]. In endometrial stromal sarcomas, Jazf1–SUZ12 rearrangement exacerbates cancer through inhibiting the PRC2 complex, thereby lowering histone methyl transferase expression [19]. Although Jazf1 is known to be involved in prostate cancer, the mechanism of interaction between Jazf1 and prostate cancer has not yet been elucidated. In this study, we demonstrated that Jazf1 contributes to prostate cancer progression through up-regulating JNK/Slug.

First, we obtained human prostate normal and cancer tissue samples from 20 patients. Prostate cancer tissue samples exhibited higher expression of Jazf1 than a normal prostate tissue from the same patient. These results indicate that Jazf1 is highly expressed in human prostate cancer patients. On this basis, we generated Jazf1-overexpressing human prostate cancer cell lines, DU145 (androgen-independent) and LNCaP (androgen-dependent). Generally, prostate cancer cells survive because of the expression of androgen. However, castration-resistance prostate cancer (CRPC) is not affected by androgen, so androgen deprivation therapy is ineffective and new therapeutic targets are needed in CRPC [5, 9]. Therefore, we generated cell lines stably overexpressing Jazf1 in both androgen-dependent LNCaP cells and -independent DU145 cells. Overexpression cell lines exhibited increased cell proliferation compared to empty vector cell lines. In addition, colony formation ability, one of cancer’s main characteristics, was enhanced in Jazf1-overexpressing cell lines [29–31]. Similarly, we established DU145 cell lines with Jazf1 knock-down using shRNA infection. Because the two overexpression cell lines showed a similar pattern, we only used androgen-independent DU145 cells for these experiments. In Jazf1 knock-down cells, cell proliferation and colony-formation ability decreased compared to that of the scrambled cell line. To confirm tumorigenesis *in vivo*, we performed a xenograft tumor formation assay in athymic nude mice. Tumor formation capacity was enhanced in Jazf1-overexpressing cells and decreased in Jazf1 knock-down cells compared to control DU145 cells. We found that Jazf1 accelerates prostate cancer progression *in vivo* and that prostate cancer cells change phenotypes, proliferation, and colony formation upon Jazf1 expression.

Based on these data, we performed additional experiments to identify the molecular mechanism through which Jazf1 acts in prostate cancer cell lines. Previously, Jazf1 was shown to inhibit nuclear receptor TR4 by means of ligand binding in hepatocytes [11]. Additionally, TR4 is a known tumor suppressor, especially for prostate tumorigenesis [32]. However, Jazf1 and TR4 downstream signaling pathways are not known. Therefore, we performed a microarray in DU145 and LNCaP cell lines. Expression of several genes was altered in both cell lines, and five candidate genes were selected by microarray. Among these genes, the expression of Slug and vimentin was increased in two cell lines overexpressing Jazf1. Slug and vimentin are related to the epithelial-mesenchymal transition (EMT) that occurs during metastasis. Several studies showed that Slug is highly expressed in a variety of cancers, including prostate cancer [33]. Slug is increased by phosphorylation of JNK in EMT of lymphatic epithelial cells and in lens epithelial cells [34, 35]. Slug promotes prostate cancer via repressing E-cadherin and enhancing the phosphorylation of JNK and AP-1 [23, 24]. Therefore, we hypothesized that Slug may be a gene downstream of Jazf1. In addition, the levels of Slug are decreased in Jazf1 knock-down cells compared to levels in scrambled cells. Next, we measured the phosphorylation of JNK/c-Jun. This phosphorylation was increased in Jazf1-overexpressing cells and decreased in Jazf1 knock-down cells. Taken together, Jazf1 increases the phosphorylation of JNK/AP-1, enhancing Slug expression to promote prostate cancer proliferation.

Prostate cancer is frequently metastatic and has a high rate of metastasis-related death. In CRPC, cancer mainly recurs as bone metastases. Thus, regulating metastasis is important in prostate cancer [36, 37]. We observed cell migration and invasion, major abilities of metastatic cells. In the cell migration assay, Jazf1-overexpressing cell lines were more migratory empty vector cell lines. Cell invasiveness was also increased in Jazf1-overexpressing cell lines. Jazf1 knock-down cells exhibited decreased cell migration and invasion compared to scrambled cells. Next, we measured the expression of EMT markers. EMT is the transition from proliferative epithelial cells to migratory and invasive mesenchymal cells. In the progression of EMT, prostate cancer cells lose cell adhesion and cell polarity capacity, and the binding between cells weakens [38, 39]. Then, levels of epithelial cell markers decrease while mesenchymal cell markers increase. Based on these results, we observed changes in levels of the epithelial marker, E-cadherin, and the mesenchymal marker, vimentin. The expression of E-cadherin was inhibited in Jazf1-overexpressing cells but promoted in Jazf1 knock-down cells. On the other hand, vimentin behaved oppositely. Jazf1 enhanced EMT by decreasing E-cadherin and increasing vimentin expression. However, EMT and metastasis involve more transcription factors and genes [20, 40–42]. Therefore, the correlation between Jazf1 and metastasis requires further study to determine a more accurate molecular mechanism.

In conclusion, our results showed that Jazf1 promotes prostate cancer progression via up-regulating JNK/Slug expression both *in vitro* and *in vivo*. Especially, the downstream signaling of Jazf1 that promotes prostate cancer was determined *in vitro*. The Jazf1 pathway, which is both androgen dependent and independent, can also be a therapeutic target of both primary prostate cancer and CRPC. Therefore, Jazf1 may be a crucial factor for prostate cancer progression, suggesting that Jazf1 is a promising new therapeutic target for prostate cancer and CRPC.

## MATERIALS AND METHODS

### Human prostate tissue sample

Human prostate tissue specimens were collected from prostate cancer patients underwent resection in the Kyungpook National University Hospital in Daegu, Korea. The diagnosis of prostate cancer was verificated by pathological outcomes. In each patient, both normal tissue and tumor tissue were collected and used to experiments.

### Cell culture

The LNCaP cell line was cultured in RPMI1640 (Gibco, Life Technologies, Grand Island, NY, USA) and DU145 cell line was cultured in Dulbecco’s Modified Eagle Medium (DMEM, Gibco, Life Technologies, Grand Island, NY, USA) supplemented with 10% fetal bovine serum (FBS, Gibco, Life Technologies, Grand Island, NY, USA) and 1% penicillin/streptomycin (P/S, Gibco, Life Technologies, Grand Island, NY, USA). All cell lines were maintained at 37°C in a 5 % CO_2_ incubator.

### Transfection

For stable expression of Jazf1 in prostate cancer cell lines, cells were seeded into 100-mm dishes. After 24 h, cells were transfected with pEGFP-N1 or pEGFP-N1-Jazf1 vector using FuGENE HD (Promega, Madison, WI, USA) transfection reagent by following the manufacturer’s instructions. To establish stable expression, the transfected prostate cancer cell lines were treated with G418 (500 µg/mL) for 7 days. Then, subcultures were treated with G418 every 3 days.

### Lentiviral production and infection

The lentiviral Jazf1 shRNA vectors for the knockdown of Jazf1 were purchased from Sigma. Oligonucleotides were cloned into the pLKO.1 lentiviral vector. HEK293T cells were co-transfected with pLKO.1-scramble or pLKO.1-Jazf1 and pMDLg/p RRE, pMD2.G, and pRSV-Rev using FuGENE HD transfection reagent (Promega). DU145 cells were infected with lentiviruses encoding shRNA using 8 µg/mL protamine sulfate (Sigma). After 48 h, cells were selected by puromycin (1 µg/mL) for 4 days, establishing a stable knockdown of Jazf1 in DU145 cells.

### Cell proliferation (CCK-8) assay

Cell proliferation was estimated using a CCK-8 assay (Cell Counting Kit-8, Dojindo Molecular Technologies, Inc.). Cells were seeded into 96-well plates (1 × 10^3^ cells/well) and incubated for 0, 1, 2, 3, and 4 days. In total, 10 µL of CCK-8 solution was added to each well and incubated for an additional 1 h at 37°C in a 5% CO_2_ incubator. The optical density (OD) of each well was measured at 450 nm using a spectrophotometer (BioTek).

### Soft agar assay

The effects of Jazf1 on colony growth were investigated in human prostate cancer cell lines. Cells (1 × 10^4^ cells/mL), suspended in RPMI1640 or DMEM supplemented with 10% FBS and 1% P/S, were added to the top layer of 0.3% agar (Top agar), over a base layer of 0.5% agar (Bottom agar). The cells were cultured at 37°C in a 5% CO_2_ incubator for 3 weeks. The number of colonies was counted using a microscope (Leica), and the diameter of each colony was measured.

### Migration and invasion assay

Migration assays were performed in 24-well transwell plates (Corning) according to the manufacturer’s instructions. Cells (2 × 10^4^ cell per well) were seeded into the upper chamber containing serum-free medium. The lower chamber was filled with complete, supplemented medium. After 24 h, cells on the opposite side of the chamber were fixed 100% methanol and stained with crystal violet (Sigma). Invasion assays were also performed in 24-well transwell plates (Corning) using Matrigel (BD Biosciences) according to the manufacturer’s instructions. Cells (2 × 10^4^ cell per well) were seeded into the covered Matrigel upper chamber containing serum-free medium. The lower chamber was filled with complete, supplemented medium. After 48 h, cells that had invaded the Matrigel were fixed 100% methanol and stained with crystal violet (Sigma). All experiments were conducted in triplicate.

### Xenograft tumor formation assay

All procedures involving animals were performed in accordance with guidelines and approval of the Kyungpook National University. Balb/c female nude mice were injected subcutaneously in the flank with 200 µL of cells (1 × 10^7^ cells) suspended in phosphate buffered saline (PBS). Approximately 5 weeks after injection, tumor tissues were extracted from the nude mice for histological analysis.

### Immunohistochemistry

The tumor tissues extracted from nude mice were fixed in 4% paraformaldehyde at 4°C overnight, embedded in paraffin, and sectioned to a thickness of 7 µm. Sections were stained with H&E, after which immunohistochemistry was performed using a primary antibody against Jazf1 (ab80329, Abcam).

### Real-time PCR

Total RNA was harvested from cells using TRIzol reagent (Invitrogen) according to the manufacturer’s instructions. cDNA was synthesized using an RT-PCR kit (TAKARA, Tokyo, Japan) and measured using qPCR with SYBR Premix EX Taq (TAKARA). To assess the expression of human *Jazf1* and proliferation marker *Ki-67*, real-time PCR was performed using a Step One Plus PCR system (Applied Biosystems, Foster City, CA, USA). The primers used here were as follows: Jazf1 F, 5′-GGA GTC GGA CAG CGA TGA GT-3′; R, 5′-GCT TCT CTT CCC CTC CAT TCA-3′; Ki-67 F, 5′-GCA CCT AAG ACC TGA ACT ATT-3′; R, 5′-TGT GCA TTA CCA GAG ACT TT-3′; β-actin F, 5′-TGA GAT GCG TTG TTACAG GAA GTC-3′; R, 5′-GAC TGG GCC ATT CTC CTT AGA GA-3′. Relative expression was quantified and normalized to the relative expression of β-actin per sample, respectively.

### Western blotting

Lysis buffer (Intron Biotechnology, Seongnam, Korea) was used for protein extraction in cells or tumor tissues. The primary antibodies were Jazf1 (ab80329, Abcam), Slug (#9585, Cell Signaling Technology), Snail (#3895, Cell Signaling Technology), Vimentin (ab92547, Abcam), E-cadherin (ab76055, Abcam), phosphor-c-Jun (Ser243) (#2994, Cell Signaling Technology), c-Jun (#9165, Cell Signaling Technology), phospho-JNK (Thr183/Tyr185) (#9251, Cell Signaling Technology), JNK (#9252, Cell Signaling Technology), and β-actin (sc-47778, Santa Cruz). Nitrocellulose membranes were incubated overnight at 4°C with primary antibodies diluted in 5% skim milk or bovine serum albumin (BSA). Secondary antibodies, anti-rabbit and anti-mouse IgG, labeled with horseradish peroxidase (HRP) (Thermo Scientific, Seoul, Korea), were diluted in 5% skim milk or BSA; nitrocellulose membranes were incubated with the secondary antibodies for 2 h at room temperature. Immunoreactive bands were visualized using electrochemiluminescence substrate (GE Healthcare, Seoul, Korea) and protein expression was visualized using DaVinci software.

### Statistical analysis

All data are presented as means ± SD of triplicate samples from at least three independent experiments. Differences between means were assessed using ANOVA; *p* ≤ 0.05 was considered statistically significant.
